# Glucagon‐Like Peptide 1 Receptor Agonists and Chronic Lower Respiratory Disease Among Type 2 Diabetes Patients: Replication and Reliability Assessment Across a Research Network

**DOI:** 10.1002/pds.70087

**Published:** 2025-01-13

**Authors:** Mitchell M. Conover, Yasser Albogami, Jill Hardin, Christian G. Reich, Anna Ostropolets, Patrick B. Ryan, Observational Health Data Sciences and Informatics (OHDSI) Research Network

**Affiliations:** ^1^ Observational Health Data Science and Informatics New York New York USA; ^2^ Observational Health Data Analytics, Johnson & Johnson Titusville New Jersey USA; ^3^ Department of Clinical Pharmacy, College of Pharmacy King Saud University Riyadh Saudi Arabia; ^4^ Real World Solutions, IQVIA Cambridge Massachusetts USA; ^5^ Department of Biomedical Informatics Columbia University New York New York USA

**Keywords:** chronic lower respiratory disease, common data model, glucagon‐like peptide 1 receptor agonists, pharmacoepidemiology, real world data, real world evidence, reliability, replicability, reproducibility, transparency

## Abstract

**Introduction:**

The aim of this study is to use observational methods to evaluate reliability of evidence generated by a study of the effect of glucagon‐like peptide 1 receptor agonists (GLP‐1RA) on chronic lower respiratory disease (CLRD) outcomes among Type‐2 diabetes mellitus (T2DM) patients.

**Research Design and Methods:**

We independently reproduced a study comparing effects of GLP‐1RA versus dipeptidyl peptidase‐4 inhibitors (DPP4‐i) on CLRD outcomes among patients with T2DM and prior CLRD. We reproduced inputs and outputs using the original study data (national administrative claims) and evaluated the robustness of results in comparison to alternate design/analysis decisions. To evaluate generalizability, we applied an analysis protocol and conducted a meta‐analysis across a research network that includes a diverse array of populations and data sources. We also produced additional analyses evaluating individual drugs within the GLP‐1RA class and CLRD outcomes.

**Results:**

We confirmed alignment of study inputs and outputs and closely reproduced effect estimates and sensitivity analyses. Adjusted effect estimates were robust to empirical calibration. Network meta‐analysis confirmed original findings but indicated weaker effects than originally published. Meta‐analysis of drugs within the GLP‐1RA class against DPP4‐i provided evidence that effects vary within the GLP‐1RA class, indicating stronger effects for exenatide and weaker effects of dulaglutide.

**Conclusions:**

This study supports and establishes the reliability of the original study by (1) producing consistent findings in a range of alternate databases and populations, (2) demonstrating effects for multiple drugs within the GLP‐1RA class, and (3) independently confirming the reproducibility of the original study and its findings. This reliability evaluation provides the beginnings of a broader framework for using standardized tools and distributed data networks to systematically interrogate the reliability of findings generated using observational data.

**Summary**

- We reproduced study inputs, outputs, effect estimates, and sensitivity analyses for an observational study of the effect of glucagon‐like peptide 1 receptor agonists (GLP‐1RA) on chronic lower respiratory disease outcomes among Type‐2 diabetes mellitus patients.
- This study supports and establishes the reliability of the original study by producing consistent findings in a range of alternate databases and populations demonstrating effects for multiple drugs within the GLP‐1RA class.
- Adjusted effect estimates were robust to empirical calibration and large‐scale propensity‐score adjustment.
- This evaluation provides a preliminary framework for using standardized tools and distributed data networks to systematically interrogate the reliability of observational study findings.
- Further work is needed to empirically evaluate the performance of the objective diagnostics that this study uses to assess reliability.

## 1 Background

Observational causal inference research using real‐world data provides an opportunity to observe medication use in a real‐world setting and to assess the risks and benefits associated with such medications in actual practice [1]. Ongoing development of observational study designs and analytic methods have equipped observational researchers with a powerful toolkit to adjust for sources of bias. Despite substantial improvements in methods and data resources used in observational research, a large number of published observational studies still conclude with the statement “Randomized trials are warranted to confirm our findings,” even in cases where the recommended randomized trial is highly unlikely to be conducted, for example, due to challenges with feasibility and/or cost. Implicit in this trend is the assumption that observational research cannot provide reliable evidence absent of confirmation with experimental research. Given that many of‐interest health research questions simply cannot be realistically assessed using experimental methods, evidence evaluation of the reliability of observational study evidence becomes critical to meaningfully apply the findings of observational research studies.

Despite concerns about the reliability of evidence generated by observational research, established practices to improve the reliability of empirical evidence (e.g., reproduction and replication) are severely underutilized, an issue which has received widespread attention over the last decade [2, 3, 4, 5, 6, 7, 8].

Observational Health Data Sciences and Informatics (OHDSI) has posited a general framework for desirable attributes of reliable evidence (Figure S1) [9]. Evidence should be (1) repeatable (same investigator using the same data and analysis produces the same results), (2) reproducible (different investigator using the same data and analysis produces the same results), (3) replicable (same analysis performed on similar data and study populations produces similar results), (4) generalizable (same analysis performed on different data and study populations produces similar results), (5) robust (different analyses performed on different or same data produces similar results), and (6) calibrated (the same analysis performed using a set of negative control outcomes produces results indicating minimal residual systematic error after adjustment and effect estimates are consistent after calibration).

Albogami et al. recently published an observational research study in *Diabetes Care* exploring the effect of glucagon‐like peptide 1 receptor agonist (GLP‐1RA) treatment on chronic lower respiratory disease (CLRD) exacerbations among patients with Type‐2 diabetes mellitus (T2DM) and pre‐existing CLRD [10]. Separate analyses of time‐to‐first CLRD inpatient admission and the count of CLRD exacerbations associated with inpatient or outpatient visits both suggested strong protective effects (HR = 0.52 [0.32, 0.85]; IRR = 0.70 [0.57, 0.87], respectively), of GLP‐1RA versus dipeptidyl peptidase‐4 inhibitors (DPP4‐i). In their conclusions, the authors interpret these findings with two key statements: “potential beneficial effects of GLP‐1RA should be considered in selection of an antidiabetes treatment regimen” and “randomized clinical trials are warranted to confirm our findings.”

In our opinion, this study represents a high‐quality analysis relative to the majority of retrospective observational causal research published using secondary health data (i.e., health data collected for non‐research purposes). The presented analysis contained multiple approaches to test validity and evaluate robustness to study parameter variations using various analytical assumptions, within the context of a comparative cohort design. However, to increase confidence in this observational study's findings, additional work is needed to reproduce the original analysis and further examine the study's reliability with respect to robustness and generalizability. First, we sought to independently *reproduce* the Albogami et al. study as closely as possible in the original study database [10]. Then we sought to evaluate the *robustness* of the Albogami et al. study findings by conducting sensitivity analyses in the IBM Commercial Claims and Encounters (CCAE) database, including sensitivity to empirical *calibration* of effect estimates using negative control outcomes [11, 12, 13, 14]. Finally, we used the OHDSI Research Network to evaluate the *generalizability* of findings produced by the original Albogami protocol applied across different secondary health databases and study populations. Through this work, we demonstrate a preliminary framework for using standardized tools and distributed data networks to reproduce and evaluate the reliability of clinical findings based on observational studies.

## 2 Methods

### 2.1 Reproducibility

In the primary analysis, we sought to reproduce, as closely as possible, the original analysis performed by Albogami et al. inclusive of all study outputs. To do so, we reviewed both the published manuscript for the study and the analytical code, which was furnished to OHDSI by the Albogami study team. To briefly summarize the original analysis, Albogami et al. conducted their study within the IBM CCAE database, which is a nationwide sample of adjudicated administrative claims from patients with commercial employer‐sponsored health insurance. Using data from 2006 to 2017, the authors conducted a retrospective new‐user cohort study comparing GLP‐1RA initiators (*N* = 4150) to an active comparator (DPP4‐i, *N* = 12 540) to assess the risk of two outcomes using a one‐year follow‐up period: (1) time‐to‐first CLRD hospitalization and (2) count of any CLRD exacerbations associated with an emergency room visit, inpatient admission, or an outpatient visit that was followed by oral steroid treatment. To identify patients with documented T2DM and CLRD, the authors required patients to have at least one inpatient or two outpatient encounters with T2DM and CLRD, based on the presence of diagnosis codes (International Classification of Diseases 9th [ICD‐9] or 10th [ICD‐10] revision) or an outpatient encounter and dispensing of relevant medications in the year before the index date.

The Albogami et al. analysis used stabilized inverse‐probability of treatment weighting (sIPTW) to balance a list of 45 potential confounders. After re‐weighting to address confounding the authors generated adjusted effect estimates using Cox proportional hazards regression to estimate the hazard ratio (HR) for the primary (time‐to‐event) outcome and Poisson regression to estimate the incidence rate ratio (IRR) for the secondary (count) outcome. For both estimates, they also produced 95% confidence intervals to quantify uncertainty. These findings were shown to be robust to several sensitivity analyses, including use of an alternate comparator (sulfonylureas). The authors also explored the use of a negative control outcome analysis (skin infection), which did not indicate the presence of residual confounding or healthy user bias.

In this reproducibility study, the analyses expanded upon the original Albogami study. While the original analysis used sIPTW to balance 45 pre‐selected potential confounders and produce an estimate of the treatment effect in the treated population, we used data‐driven large‐scale regularized logistic regression in conjunction with propensity score (PS) stratification (using five quintiles defined within the GLP‐1 exposed population) to balance 20 036 covariates including demographics, prior/comorbid conditions, drug exposures, procedures, and health‐service‐use behaviors. Additionally, we used outcome models (Cox and Poisson for the primary time‐to‐event and secondary count outcomes respectively), which were conditioned on the PS strata or PS‐matched pairs.

We reproduced several sensitivity analyses, which Albogami et al. conducted to evaluate the robustness of findings related to their primary CLRD hospitalization outcome. There were four sensitivity analyses included in the original publication which were not reproduced in this study: (1) an analysis that used a Bayesian additive regression tree (BART) approach to estimate the PS, (2) an analysis that used multiple imputation for missing obesity and smoking covariates, (3) an analysis that used inverse‐probability‐of‐censoring weighting (IPCW) to account for differential loss‐to‐follow‐up, and (4) an analysis restricted to patients who had GLP‐1RA or DPP4‐i treatments added to prevalent metformin therapy. These analyses may indeed be informative but were not feasible within the scope of this study. On the other hand, we conducted four additional sensitivity analyses that were not in the original publication, including (1) a 1:100 variable‐ratio PS matching analysis using a conditional Cox outcome model, (2) an analysis relaxing the requirement that the GLP‐1RA or DPP4‐i therapy be added on to an active prior diabetes therapy to only require that a prior diabetes therapy occurred within the year prior to GLP‐1RA or DPP4‐i initiation, and (3) a cumulative time analysis that expanded the study time period to include all currently available data (extending the end of the study from 2017 to 2020), and (4) a disjoint time analysis which separately studied the periods 2006 to 1 October 2014 and 1 November 2015to 2020. The disjoint time analysis was motivated by a desire to separately analyze the periods where outcomes were classified using ICD‐9 versus ICD‐10 codes. In this sub‐analysis, patients with index dates between 1 October 2014 and 1 November 2015 were dropped since their follow‐up would include time were outcomes were classified by a mix of ICD‐9 versus ICD‐10 codes.

Finally, we also conducted a sensitivity analysis, which specified study cohorts using OHDSI standards (i.e., using OMOP Standardized Vocabularies to define clinical concepts). This sensitivity analysis is particularly relevant since the use of OHDSI standards relaxes the dependence on the original study data source (IBM CCAE) and allows the study to be executed within multiple data sources in the OHDSI Research Network, which are all mapped to OHDSI Common Data Model (CDM) [15].

### 2.2 Generalizability

The IBM CCAE database was used to evaluate reproducibility, since that was the database used in the original study published by Albogami et al. In order to evaluate the generalizability of the Albogami findings, we executed the analysis within five additional secondary healthcare databases, which are described in Table S1. For each data resource, we provide a brief description and size of the population it represents as well as the calendar years and data that it captures. These additional databases include (1) IBM MarketScan Medicare Supplemental Database (IBM MDCR), (2) IBM MarketScan Multi‐State Medicaid Database (IBM MDCD), (3) Optum ClinFormatics Data Mart (Optum ClinFormatics), (4) Optum Electronic Health Records (Optum EHR), and (5) IQVIA Adjudicated Health Plan Claims (IQVIA PharMetrics Plus).

In order to further assess the robustness of estimates generated by the single analysis within a single data source, the following additional diagnostics were performed: power calculations estimating minimum detectable relative risk; preference score distributions (a transformation of PS distributions that adjusts for prevalence differences between populations) to evaluate empirical equipoise and test assumptions of positivity and limited confounding [16, 17]; patient characteristics to evaluate covariate balance before and after PS‐adjustment as measured by the absolute standardized mean difference (ASMD) an indicator of confounding [18, 19, 20, 21]; Kaplan–Meier plots to examine proportionality assumptions in the analyses estimating the hazard ratio, and negative‐control calibration plots showing effect estimates corresponding to a large set of negative control outcomes to assess residual systematic bias. Effect estimates generated by analyses that pass diagnostic inspections were aggregated across data sources using a random effects meta‐analysis model. Before combining into a meta‐analytic estimate, we also inspected effect estimates for cross‐database heterogeneity as indicated by the *I* ^2^ score, which quantifies the proportion of total variation across studies that is due to heterogeneity rather than chance [22].

### 2.3 Confidence‐Interval Calibration

For each effect estimate generated (i.e., for each comparison within each database), we fit an empirical null distribution reflecting the distribution of adjusted effect estimates corresponding to up to 60 negative‐control outcome experiments (each of which were required to reflect at least five cases in order to be included). This distribution collectively reflects both random and residual systematic error after adjustment. The empirical null distribution can be used to identify sources of systematic bias and, with empirical calibration, correct effect estimates and confidence intervals [11, 12, 13, 14].

### 2.4 Effects of Specific GLP‐1RA Drugs

Finally, the effect described by Albogami et al. described that of the entire GLP‐1RA class as there was insufficient power in the original study to stratify by individual drugs within the class. Given the added power afforded by inclusion of multiple data sources in the OHDSI Research Network and additional data years (2006–2020), we studied the effects and prescribing trends of four GLP‐1RA drugs separately (exenatide, liraglutide, dulaglutide, and semaglutide) using random effects models to meta‐analyze estimates generated from the individual databases.

### 2.5 Open and Transparent Science

In keeping with a philosophy of open and transparent science, the work conducted on this project and the generated results have been shared (including open‐source, executable code available here: https://github.com/ohdsi‐studies/Glp1ClrdEstimation) such that investigators with access to data resources formatted to the OMOP CDM, can independently execute analyses to confirm our findings and their reliability.

## 3 Results

For all analyses, we produced a priori study diagnostics, which were assessed before inspecting effect estimates and used to inform interpretation of the results. Study diagnostics for evaluations of the CLRD hospitalization outcome in the IBM CCAE database are available here: https://data.ohdsi.org/GLP1ReproducibilityHospitalization/. Study diagnostics for evaluations of the CLRD exacerbation outcome in the IBM CCAE database are available here: https://data.ohdsi.org/GLP1ReproducibilityExacerbation/. Lastly, study diagnostics for the evaluations run across the OHDSI Research Network are available here: https://data.ohdsi.org/GLP1Generalizability/. Study diagnostics for primary comparisons are shown in Table S2. A guide to using the web‐based Evidence Explorer application to view results is provided in Supporting Information.

### 3.1 Reproducibility

First, we observed extremely close alignment with Albogami et al. reproducing the original exposure cohorts being compared. In the IBM CCAE database, our reproducibility analysis produced exposure cohorts that were nearly identically sized compared to the original Albogami et al. analysis. In our reproducibility study, we identified 4315 GLP‐1RA initiators and 12 517 DPP4‐i initiators, while the original Albogami et al. study identified 4150 GLP‐1RA initiators and 12 517 initiators respectively. A more detailed inspection of attrition which breaks out the impact of each study eligibility criterion also showed nearly identical alignment (Figure S2, Table S3).

Relative to the Albogami et al. study, our reproducibility analysis showed nearly perfect alignment of baseline covariate distributions (before adjustment) within the GLP‐1RA and DPP4‐i cohorts with respect to gender, patient age, depression, dyslipidemia, hypertension, obesity, pneumonia, and congestive heart failure (Table S4). The largest absolute percentage difference was observed in the baseline prevalence of obesity, which was only 1.6% for GLP‐1RA cohorts versus 1.8% for DPP4‐i cohorts. For all other covariates, absolute percentage differences were < 1%.

In Table 1, we show comparisons of the follow‐up statistics, outcome counts, and incidence rates among initiators of GLP‐1RA and DPP4‐i in the original Albogami et al. study, in our IBM CCAE reproducibility study, and across the OHDSI Research Network. In the original study database (IBM CCAE), relative to Albogami et al., our reproducibility study identified fewer CLRD hospitalization outcomes in both exposure groups and identified more CLRD exacerbation outcomes in both exposure groups. We aligned closely on estimates of mean days and total person‐years of follow‐up. Across the OHDSI Research Network, the analysis generated wide ranging estimates of outcome incidence which may result from population heterogeneity, data capture processes, and/or care delivery.

**TABLE 1:** Comparison of subject counts and outcome incidence rates per 1000 person‐years (PY) in the original Albogami et al. study and the OHDSI reproducibility study.

	Target: GLP‐1RA new users			Comparator: DPP4‐i new users		
Database	Subjects	Hospitalization incidence rate (/1000 PY)	Exacerbation incidence rate (/1000 PY)	Subjects	Hospitalization incidence rate (/1000 PY)	Exacerbation incidence rate (/1000 PY)
Reproducibility analysis						
IBM CCAE: Albogami	4150	11.0	58.2	12 540	20.6	71.5
IBM CCAE: OHDSI	4027	6.91	71.04	12 473	14.52	85.24
OHDSI network analysis						
IBM CCAE (w/addl. years)	8583	4.91	76.53	16 539	11.84	87.71
Optum ClinFormatics	6911	42.6	164.09	15 164	73.36	195.63
IBM MDCD	2766	29.14	284.81	6867	54.46	354.12
IBM MDCR	971	26.84	129.26	7110	48.56	172.8
Optum EHR	924	< 11.73	79.7	1792	23.33	79.96
IQVIA PharMetrics	7522	9.98	123.65	8293	13.95	130.35

We produced a diagnostic plot intended to show covariate balance between the two groups being compared, before and after adjustment (Figure S3) since residual covariate imbalance may indicate bias in adjusted effect estimates. Similar to the original Albogami et al. study, our covariate balance diagnostic indicates that while some covariates were imbalanced between the crude GLP‐1RA and DPP4‐i cohorts, no covariate exceeded an ASMD of 0.10 after PS‐stratification was applied, which indicates minimal confounding (with respect to measured covariates) in adjusted effect estimates [18, 19, 20, 21].

**FIGURE 1:**
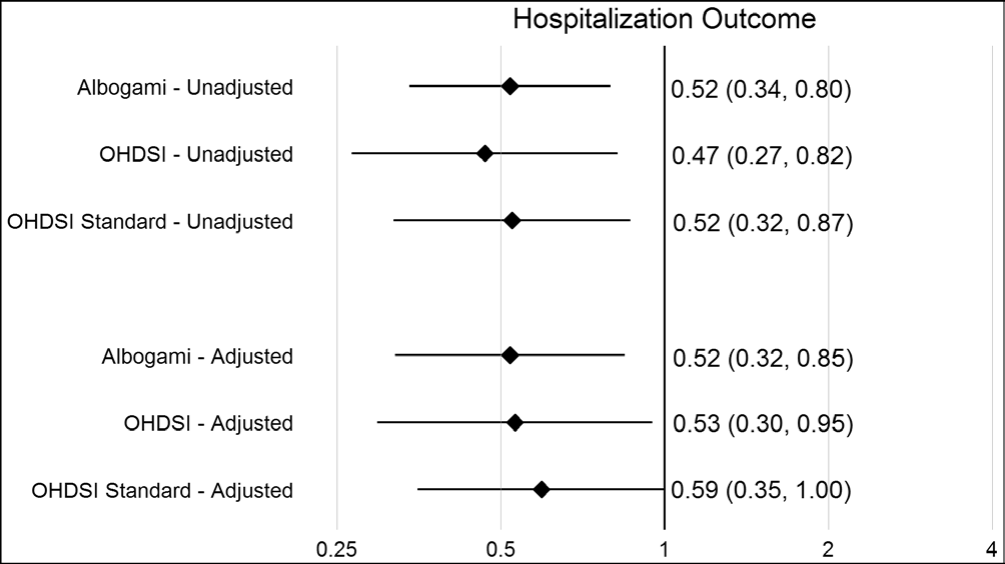
Agreement of effect estimates between Albogami et al., OHDSI reproducibility study, and OHDSI standardized cohorts, for the primary CLRD hospitalization outcome (top panel) and the secondary CLRD exacerbation count outcome (bottom panel).

Figure 1 below shows unadjusted and adjusted effect estimates for both the hospitalization and exacerbation outcomes from the original Albogami study and the OHDSI reproducibility study. Despite aforementioned differences in outcome ascertainment, effect estimates from the reproducibility evaluation were highly concordant (i.e., all confidence intervals contained the point estimates of all effects), regardless of whether we used exposure and outcome cohorts that were deliberately aligned with the original Albogami analysis or cohorts that implemented OHDSI Standard Vocabulary and network‐compatible data specifications. For the purpose of brevity, subsequent results will all reflect findings produced using cohorts that implement OHDSI‐standards.

In order to further inform our interpretation of these effect estimates, we used large set of negative control outcomes to generate an empirical null distribution, which can be used to inspect each analysis for residual systematic bias. Figure S4 shows the plot for the primary analysis in the IBM CCAE database. Both the calibrated and uncalibrated plots show a well‐centered distribution around the null (i.e., the true effect estimate for negative control outcomes), indicating minimal residual systematic error, regardless of whether empirical calibration is accounted for in effect estimates. Given that the original Albogami et al. analysis only used a single negative control outcome in a sensitivity analysis, demonstrating that the Albogami et al. finding is robust to empirical calibration using a large set of negative control outcomes adds substantial confidence.

As shown in Figure S5, we reproduced and confirmed a range of sensitivity analyses completed as part of the original study publication. We also examined the robustness of findings to the years of data considered. First, the cumulative time analysis including three additional years (2018–2019) not included in the Albogami et al. study, produced estimates of similar strength and magnitude for both the hospitalization (HR = 0.57 [0.36, 0.93]) and the exacerbation outcomes (HR = 0.82 [0.70, 0.90]). In contrast, the analysis of disjoint time was a rare case where findings were not consistent and robust. Analyses restricting to the earlier years of data (2006 to 1 October 2014) produced effect estimates that were stronger than the original analysis for both hospitalization (HR = 0.38 [0.18, 0.80]) and exacerbation outcomes (HR = 0.51 [0.39, 0.66]). Analyses restricted to more recent years of data (1 November 2015 to 2020) produced null effect estimates for both hospitalization (HR = 1.33 [0.58, 3.02]) and exacerbation outcomes (HR = 0.95 [0.79, 1.15]). Changing patterns in the specific GLP‐1RA drugs being prescribed between these periods (Figure S6), where initial prescribing of exenatide and liraglutide shifted to more frequent prescribing of dulaglutide and semaglutide, offers one potential sources of the observed heterogeneous effects.

### 3.2 Generalizability

We used the OHDSI‐standardized cohorts to run analyses of available databases in the OHDSI Research Network potentially spanning from 2006 to 2020. Analyses conducted in the IBM MDCR and Optum EHR databases both were under‐powered, with limited GLP‐1RA exposures (*N* < 1000) in both databases. The limited available patient sample reduced the ability of the PS‐methods to meaningfully balance covariates, resulting in a non‐negligible number of covariates presenting with ASMDs between 0.10 and 0.20 after adjustment (including potentially important confounders such as patient age and calendar year). Furthermore, the limited sample size limited the number of negative control outcomes that could be used to inform the empirical null distribution. This makes it challenging to understand whether meaningful systematic error is biasing the adjusted effect estimates generated in these databases. Despite these findings, we decided to include effects from these databases in our random‐effects meta‐analyses.

**FIGURE 2:**
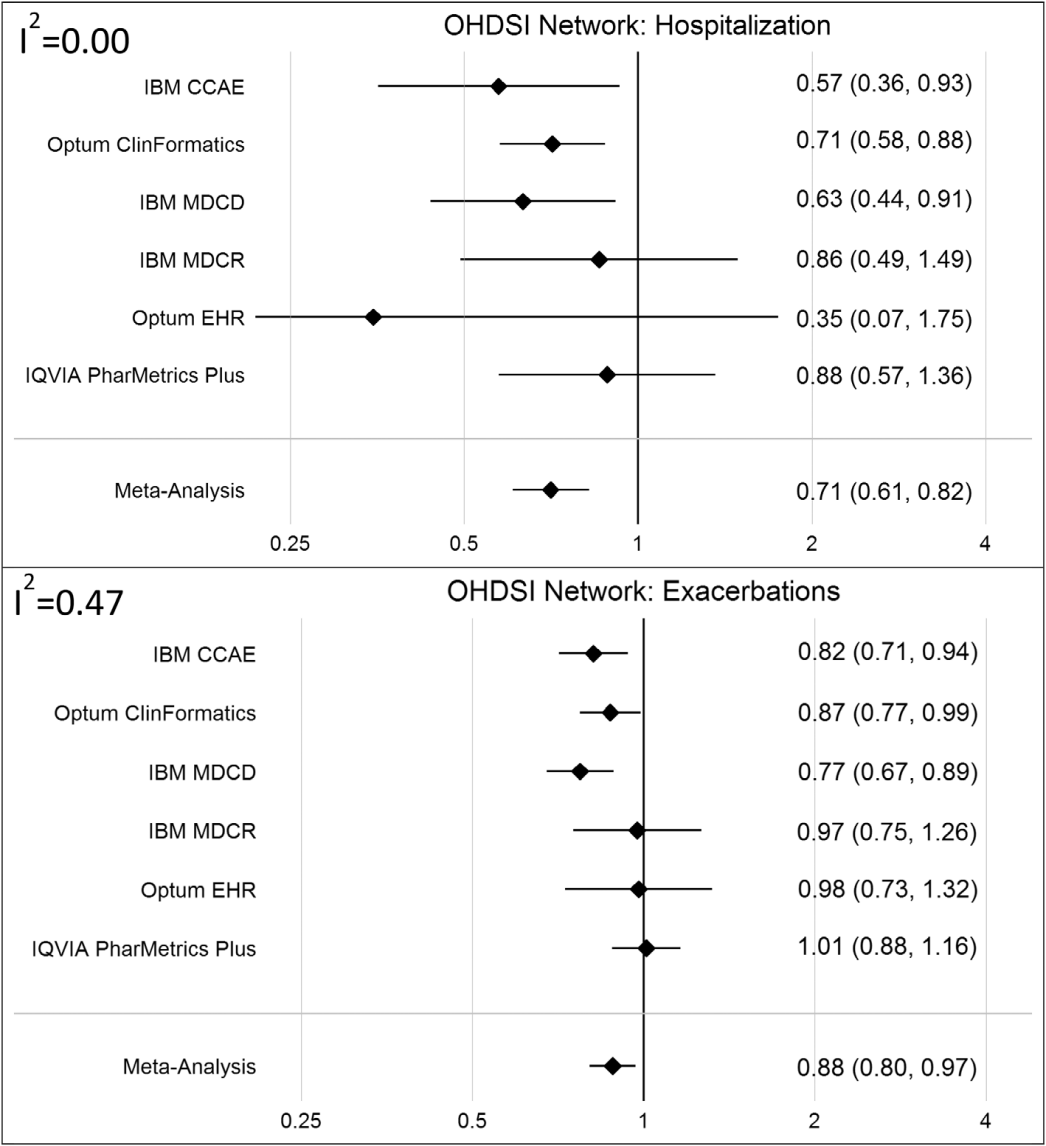
Effect estimates generated by OHDSI standardized cohorts and analyses executed across the OHDSI Research Network, including summary effects pooled using a random‐effects meta‐analysis with the corresponding *I* ^2^ value as an indicator of effect heterogeneity before pooling for the primary CLRD hospitalization outcome (top panel) and the secondary CLRD exacerbation count outcome (bottom panel).

As shown in Figure 2, we observed greater heterogeneity in effect estimates across databases, for both the CLRD hospitalization outcome and the CLRD exacerbation count outcomes. The *I* ^2^ statistic, which indicates effect estimate heterogeneity was 0.00 for the hospitalization outcome; however this may be partially driven by the wide confidence intervals around these estimates. The *I* ^2^ statistic was 0.47 for the exacerbation outcome as the effect was observed to be weaker in some database. It is important to note that the diagnostic assessments of analyses in the IBM MDCR and Optum EHR databases indicated potentially problematic covariate imbalance. Furthermore, the empirical null distribution for the analysis in the Optum EHR database indicated some residual systematic bias. Thus, these estimates should be interpreted with caution. Meta‐analyses of results across these data support the original findings from Albogami et al., though they do appear to indicate a less pronounced effect than was described in the original publication.

**FIGURE 3:**
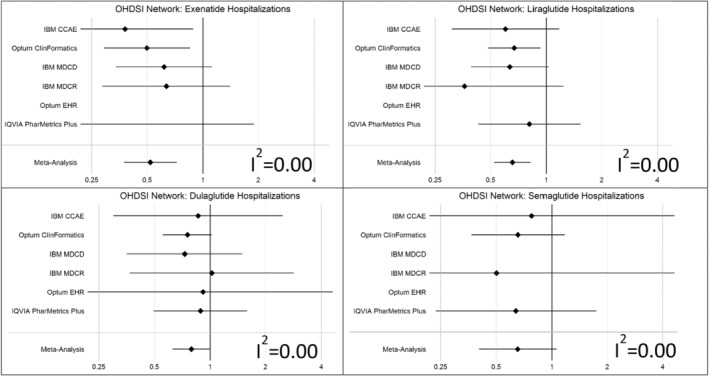
Effect estimates stratified by drug (top‐left panel: exenatide, top‐right panel: liraglutide, bottom‐left panel: dulaglutide, bottom‐right panel: semaglutide) within the GLP‐1RA class (compared to DPP4‐i class) for analyses executed across the OHDSI Research Network (2006–2020), including summary effects pooled using a random‐effects meta‐analysis with the corresponding *I* ^2^ value as an indicator of effect heterogeneity before pooling for the primary CLRD hospitalization outcome.

The OHDSI Network analysis (Figures 3 and S7) comparing specific GLP‐1RA drugs against the DPP4‐i class provided some evidence that effects vary within the GLP‐1RA class. Notably, meta‐analysis effect estimates indicate a more pronounced effect for exenatide and a slightly weaker effect of dulaglutide. Point estimates were all indicative of protective effects, however meta‐analysis effect estimates were nonsignificant for dulaglutide and semaglutide.

## 4 Discussion

This study represents a robust examination of the reliability of the findings by Albogami et al. that GLP‐1RAs have a protective effect on CLRD outcomes and provides meaningful new evidence supporting the finding. We used the OHDSI Research Network and standard tools and meta‐analysis to (1) expand the generalizability of the finding in meta‐analyses including a more diverse array of study populations (older, younger, commercially‐insured, limited‐income) and database types (administrative claims and EHR), (2) demonstrate that the effect is observable for multiple drugs within the GLP‐1RA class, and (3) independently confirm the reproducibility of the Albogami et al. study and its findings including all study inputs and outputs.

From its inception, the declared goal of the OHDSI community has been to establish an international collaborative by building on open‐science values [9]. This work to evaluate the reliability of the Albogami et al. study directly aligns with OHDSI's open‐science strategy and its commitment to enable more meaningful collaboration in the health research community. OHDSI has developed a broad toolkit to make such work possible, including the use of open‐source software, public availability of all conference proceedings and materials, and transparent, open‐access publication of generated medical evidence. These developments enable the OHDSI community and users of its open‐source tools to conduct high‐impact observational health research, and to advance clinical understanding of the reliability of existing health research. Evaluating reliability of observational evidence highlights the importance of transparency and complete study reporting, which are critical to generating reproducible evidence [23, 24, 25]. This work represents an early step toward a more robust system of evaluating evidence quality. The OHDSI website (https://ohdsi.org/) provides additional information about how to use OHDSI's open‐source tools, and identify opportunities for research collaboration.

In the original publication, Albogami et al. concluded that “potential beneficial effects of GLP‐1RA should be considered in selection of an antidiabetes treatment regimen” but added that “randomized clinical trials are warranted to confirm our findings.” However, this more exhaustive evaluation has substantially strengthened the evidence and confirmed the reliability of Albogami et al.'s findings. Furthermore, a randomized clinical trial studying CLRD outcomes among patients treated with GLP‐1RA drugs is unlikely to be conducted in the near‐future. Thus, in absence of clearer evidence, we propose that these findings be incorporated into treatment guidelines informing selection of antidiabetes treatments. Specifically, we propose that clinicians treating patients with T2DM and a history of CLRD consider use of GLP‐1RA in cases where there is not a strong motivating reason to select another therapy.

We acknowledge the possibility that methodological limitations may exist that systematically bias both the original and this reproducibility study, potentially yielding the same erroneous findings in both. When feasible, this reproducibility analysis incorporates additional evaluations of various sources of systematic error. For example, unmeasured confounding is an important threat to all observational research studies. However, we increase confidence by incorporating additional analyses which we believe reduce concerns related to unmeasured confounding: (1) assessing a large set of negative control outcomes using the empirical null distribution and calibrating effect estimates, and (2) using large‐scale PS adjustment, (3) evaluating covariate balance across a substantially larger set of covariates [11, 12, 13, 14, 26, 27, 28, 29, 30]. Prior work has demonstrated that large‐scale PS adjustment can account for unmeasured confounding when covariates are correlated or pinpointed by measured covariates, although other variables that are not correlated with any of the observed factors could induce bias [30]. Additionally, we may worry that the protective effects we observe are due to more intense surveillance for CLRD outcomes among DPP4‐i patients. However, if that were the case we would expect the increased medical surveillance to systematically bias the empirical null distribution analysis, which appears to be well‐centered with empirical calibration having relatively little impact. While these analyses cannot entirely eliminate concerns related to systematic bias they are intended to reduce them and to increase confidence in the evidence.

There are several important limitations to this reliability evaluation that we would like to highlight. First, we did encounter challenges in our evaluation of reproducibility, particularly with respect to outcome incidence rates. Our results do not indicate any systematic issue with under‐ or over‐ascertainment of outcomes, given that our reproducibility generated a lower outcome count for the primary outcome of interest (first CLRD hospitalization) and a higher incidence rate for the secondary outcome of interest (CLRD exacerbation count). Regardless of cause, the differences observed in outcome incidence between the original Albogami study and the OHDSI reproducibility study had minimal impact on effect estimates (Figure 1) which were consistent with Albogami et al. Second, we did not explore two additional avenues that would have allowed us to further interrogate the reliability of these findings: (1) running analyses in study populations outside the United States and (2) using alternate study designs (e.g., self‐controlled observational study designs) to confirm the finding. Third, in our generalizability evaluation we did observe some heterogeneity when estimating effects in different databases. In the IQVIA PharMetrics Plus we observed null effects in the analysis of the entire GLP‐1RA class. This heterogeneity may be partially explained by the fact that those data capture a different data range (post‐2015) than the other databases included and thus reflect greater dominance of one GLP‐1RA drug (dulaglutide). The latter showed some evidence of a weaker effect relative to other GLP‐1RA drugs that were more frequently prescribed in earlier calendar years. Fourth, time varying confounding (e.g., due to informative right‐censoring in the as‐treated exposure analysis) may bias our findings. We note that the original Albogami et al. study addressed this limitation in a sensitivity analysis applying IPCW (generating results consistent with the main analysis) but may not have fully accounted for informative censoring due to unmeasured confounders. Lastly, our application of the negative control distribution relies on an assumption of exchangeability between the negative controls and the drug‐outcome hypothesis of interest. Violating the exchangeability assumption may limit the ability of the negative control distribution to calibrate estimates and identify residual systematic error [11, 28].

In summary, we have conducted a broad evaluation and confirmed Albogami et al.'s findings. In scenarios such as this, where randomized clinical trials are infeasible and unlikely to be conducted, we assert that observational evidence must be used in a more meaningful way to improve patient treatment outcomes. Further work is needed to empirically evaluate the performance of the objective diagnostics that this study uses to assess reliability. However, we believe this reliability evaluation provides the beginnings of a broad framework for using standardized tools and the diverse patient populations and data types captured in distributed data networks to more thoroughly and systematically interrogate the reliability of clinical findings generated using observational data.

## Disclosure

Sponsors: Observational Health Data Sciences and Informatics (OHDSI) Research Network, Janssen Research & Development, a Johnson & Johnson Company.

## Conflicts of Interest

Four of the co‐authors for this work (M.M.C., J.H., A.O. and P.B.R.) are employed by Janssen Research & Development LLC, a Johnson & Johnson company, which markets products that are used to treat Type‐2 diabetes mellitus; however, those products are not evaluated in this study.

## Supporting information


Data S1.

## Data Availability

The work conducted on this project and the generated results have been shared (including open‐source, executable code: https://github.com/ohdsi‐studies/Glp1ClrdEstimation) such that investigators with access to data resources formatted to the OMOP CDM can independently execute analyses to confirm our findings and their reliability. The repository also contains documentation of study cohorts and a protocol published before the conduct of the study.
