# Supplementary material for: Glucagon‐Like Peptide 1 Receptor Agonists and Chronic Lower Respiratory Disease Among Type 2 Diabetes Patients: Replication and Reliability Assessment Across a Research Network
Source: Pharmacoepidemiol Drug Saf. 2025 Jan 13;34(1):e70087. doi: 10.1002/pds.70087 (PMC11730806; doi:10.1002/pds.70087)
Supplement: Supplementary file 1 — Data S1. [file PDS-34-e70087-s001.docx]

# Supplemental Appendix: Supplemental Content

**Guide to using the web-based Evidence Explorer application to view results.**

The web-based Evidence Explorer application presents the following information for all combinations of target, comparator, outcome, data source, and analytical adjustment approach: (1) adjusted hazard ratios (before and after calibration), (2) study power statistics, (3) cohort attrition diagrams, (4) population characteristics before and after adjustment, (5) propensity model variables and beta coefficients, (5) propensity scores plots, (6) covariate balance before and after adjustment (for all covariates), (7) systematic error evaluated using the empirical null distribution, and (7) Kaplan-Meier plots. In order to view results, drop-down menus can be used to select relevant combinations of target (GLP-1RA) and comparator (DPP4-i) cohorts that were evaluated in this study. For example, in the CLRD exacerbation dashboard ([LINK](https://data.ohdsi.org/GLP1ReproducibilityExacerbation/)), the relevant combinations of target and comparator cohorts are as follows:

| **Target Cohort** | **Comparator Cohort** |
| --- | --- |
| GLP1 new users via source codes 2006-2017 | DPP4 new users via source codes 2006-2017 |
| GLP1 new users via standard concepts 2006-2017 | DPP4 new users via standard concepts 2006-2017 |
| GLP1 new users 2006-2020 | DPP4 new users 2006-2020 |
| GLP1 new users 2006-1Oct2014 with ICD9CM | DPP4 new users 2006-1Oct2014 with ICD9CM |
| GLP1 new users 1Nov2015-2020 with ICD10CM | DPP4 new users 1Nov2015-2020 with ICD10CM |

# Supplemental Appendix: Supplemental Figures


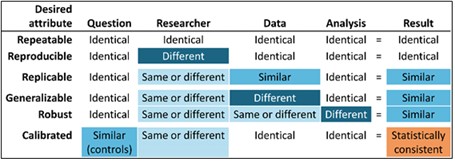


**Figure S1**. Desired attributes of reliable evidence (*source:* [*Book of OHDSI*](https://ohdsi.github.io/TheBookOfOhdsi/EvidenceQuality.html))

* For ease of presentation, these bar charts show attrition agreement after the first eligibility criterion (requiring CLRD diagnosis in the prior 365 days) was already applied. Agreement before that criterion was 231,459 and 220,073 for Albogami and OHDSI GLP-1RA cohorts (respectively) and 424,171 and 392,110 patients for the Albogami and OHDSI DPP4-i cohorts (respectively).

**Figure S2**. Attrition agreement for selection of target and comparator cohorts in original Albogami et al study and OHDSI reproducibility analysis within the IBM CCAE database


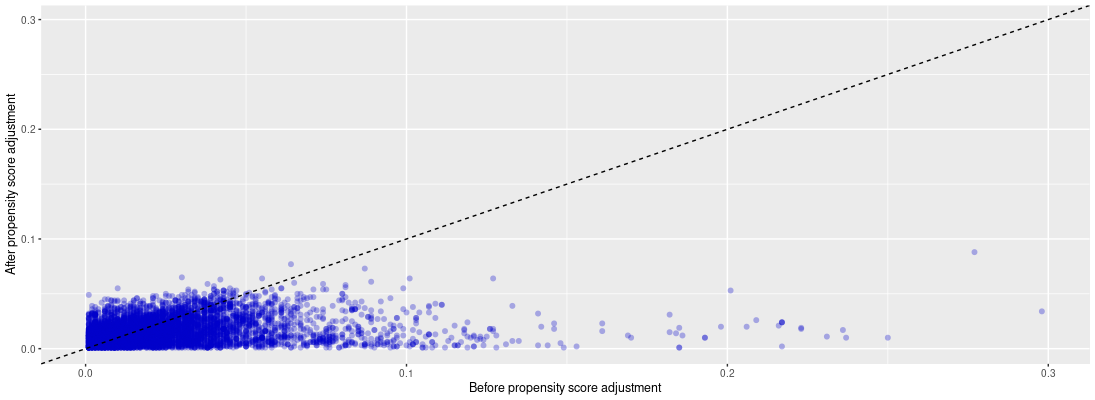


**Figure S3.** Covariate balance before and after propensity score adjustment. Each dot represents the absolute standardized difference of means (ASDM) for a single covariate before and after propensity score stratification (5 quintiles defined in the exposed population). As shown, all covariates produce ASDM < 0.10 after statistical adjustment.


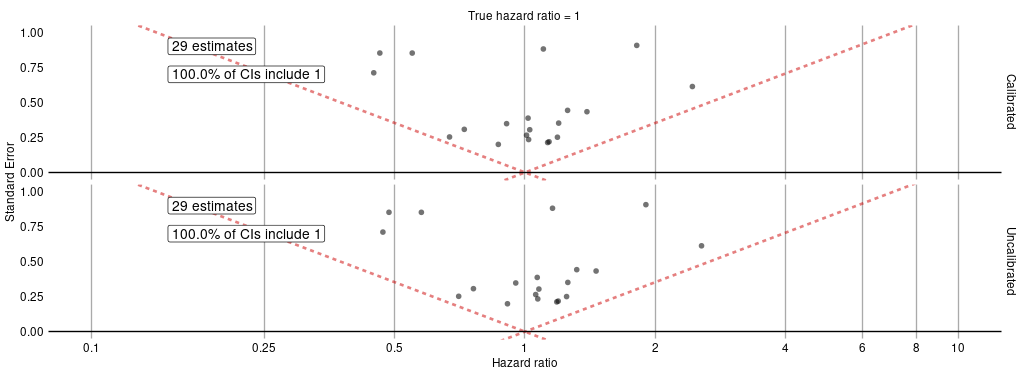


**Figure S4.** Empirical null distribution showing adjusted effect estimates for 29 sufficiently-powered negative control outcomes (out of 60 considered) before (bottom panel) and after (top panel) empirical calibration.

| No empirical calibration of confidence intervals | Empirical calibration of confidence intervals |
| --- | --- |
|  |  |

**Figure S5**. Effect estimates for sensitivity analyses completed as part of the OHDSI reproducibility study before and after empirical calibration

**Figure S6.** Annual prescribing of individual drugs within the GLP-1RA class over the study period

|  |  |
| --- | --- |

**Figure S7.** Effects of individual drugs within the GLP-1RA class (exenatide, liraglutide, dulaglutide and semaglutide) compared to the entire DPP4-i class combined across the OHDSI Research Network using random-effects meta analysis.

# Supplemental Appendix: Supplemental Tables

**Table S1.** Data sources used to reproduce and generalize study findings from Albogami et al.

| **Data source** | **Population** | **Patients** | **History** | **Data capture and short description** |
| --- | --- | --- | --- | --- |
| IBM MarketScan Commercial Claims and Encounters (IBM CCAE) | Commercially insured, < 65 years (U.S.) | 142M | 2000 – | Adjudicated health insurance claims (e.g. inpatient, outpatient, and outpatient pharmacy) from large employers and health plans who provide private healthcare coverage to employees, their spouses and dependents. |
| IBM MarketScan Medicare Supplemental Database (IBM [MDCR](https://catalog.rwe.jnj.com/index#jnjsearches?dataSetUri=%2Fdataset%2F87883da0-c84d-4315-ae1c-b3e4f51cfe1d.xml)) | Commercially insured, 65$+$ years (U.S.) | 10M | 2000 – | Adjudicated health insurance claims of retirees with primary or Medicare supplemental coverage through privately insured fee-for-service, point-of-service or capitated health plans. |
| IBM MarketScan Multi-State Medicaid Database (IBM [MDCD](https://catalog.rwe.jnj.com/index#jnjsearches?dataSetUri=%2Fdataset%2F4136471c-0662-4ca7-b091-4d6120358f74.xml)) | Medicaid enrollees, racially diverse (U.S.) | 26M | 2006 – | Adjudicated health insurance claims for Medicaid enrollees from multiple states and includes hospital discharge diagnoses, outpatient diagnoses and procedures, and outpatient pharmacy claims. |
| Optum Clinformatics Data Mart (Optum Clinformatics) | Commercially or Medicare insured (U.S.) | 85M | 2000 – | Inpatient and outpatient healthcare insurance claims. |
| Optum Electronic Health Records (Optum EHR) | General population (U.S.) | 93M | 2006 – | Clinical information, prescriptions, lab results, vital signs, body measurements, diagnoses and procedures derived from clinical notes using natural language processing. |
| IQVIA Adjudicated Health Plan Claims (PharMetrics Plus) | Commercially or Medicare insured (U.S.) | 102M | 2015 – | Inpatient and outpatient insurance claims, prescription and office/outpatient administered drugs, costs and enrollment information. |

**Table S2.** Database diagnostics for comparisons of GLP-1RA vs. DPP4-i initiation in the six study databases

| **Database** | **Data** | **Population** | **Time** | **Clinical equipoise** | **Covariate balance** | **Empirical null** |
| --- | --- | --- | --- | --- | --- | --- |
| IBM CCAE | Closed claims | US privately insured | 2006-2020 | **Pass** | **Pass** | **Pass** |
| Optum ClinFormatics | Closed claims | US privately insured + Medicare supplemental | 2007-2020 | **Pass** | **Pass** | **Pass** |
| IBM MDCD | Closed claims | US state insurance for low-income | 2006-2020 | **Pass** | **Pass** | **Pass** |
| IBM MDCR | Closed claims | US retirees with Medicare supplemental benefit | 2006-2020 | **Pass** | **Fail** | **Pass** |
| Optum EHR | Electronic health records | US received care in health provider organizations | 2006-2020 | **Pass** | **Fail** | **?** |
| IQVIA PharMetrics Plus | Closed claims | US privately insured + Medicare supplemental | 2016-2020 | **Pass** | **Pass** | **Pass** |

Clinical equipoise was evaluated by a visual assessment of the overlap of the preference score distributions. Covariate balance was evaluated by confirming that all assessed covariates were balanced between the two exposure groups with ASMD<0.10. The empirical null distribution was also visually assessed by confirming the central tendency of the negative control distribution.

**Table S3**. Attrition agreement for selection of target and comparator cohorts in original Albogami et al study and OHDSI reproducibility analysis within the IBM CCAE database

|  | **GLP-1RA** | | **DPP4-i** | |
| --- | --- | --- | --- | --- |
| **Inclusion Rules** | **Albogami** | **OHDSI** | **Albogami** | **OHDSI** |
| GLP-1RA or DPP4 users | 255,000 | 244,832 | 460,000 | 428,965 |
| With CLRD diagnosis | 23,541 | 24,759 | 35,829 | 36,855 |
| No excluded medical conditions | 18,021 | 19,184 | 28,127 | 29,630 |
| No prior exposure to alternate exposure | 13,845 | 14,557 | 26,655 | 27,674 |
| No prior exposure to insulin | 6,842 | 7,405 | 23,470 | 24,304 |
| No monotherapy users | 4,150 | 4,315 | 12,540 | 12,517 |

**Table S4.** Prevalence and absolute standardized mean difference (ASMD) comparison for key baseline covariates before analytic adjustment in the original Albogami et al study and the OHDSI reproducibility study

|  | **GLP-1RA** | | **DPP4-i** | | | **ASMD** | | |
| --- | --- | --- | --- | --- | --- | --- | --- | --- |
| **Covariate** | **Albogami** | **OHDSI** | **Albogami** | **OHDSI** | **Albogami** | | **OHDSI** |  |
| Gender: female (%) | 64.6 | 64.8 | 55.1 | 55.8 | 0.19 | | 0.18 |  |
| Age (years) | 52.0 | 52.5 | 54.3 | 54.7 | 0.28 | | -0.28 |  |
| Depression (%) | 16.2 | 16.9 | 12.4 | 12.0 | 0.10 | | 0.14 |  |
| Dyslipidemia (%) | 66.8 | 67.2 | 68.0 | 67.3 | 0.02 | | 0.00 |  |
| Hypertension (%) | 70.0 | 70.6 | 70.6 | 69.4 | 0.01 | | 0.03 |  |
| Obesity (%) | 33.6 | 32.1 | 21.0 | 19.2 | 0.28 | | 0.30 |  |
| Pneumonia (%) | 7.4 | 7.2 | 8.4 | 8.3 | 0.04 | | -0.04 |  |
| Congestive heart failure (%) | 5.5 | 5.4 | 5.4 | 5.4 | 0.00 | | 0.00 |  |

Presented covariates reflect those included in the original Abogami et al paper which are also included in the standard OHDSI population characteristics summary which can be viewed in the linked web applications. All covariates rely on a 365-day look-back window.
